# The gut-liver axis in cholangiopathies: focus on bile acid based pharmacological treatment

**DOI:** 10.1097/MOG.0000000000000807

**Published:** 2022-01-15

**Authors:** Marica Cariello, Raffaella M. Gadaleta, Antonio Moschetta

**Affiliations:** a INBB, National Institute for Biostructures and Biosystems, Rome; b Department of Interdisciplinary Medicine, ‘Aldo Moro’ University of Bari, Bari, Italy

**Keywords:** bile acid, cholangiopathy, farnesoid X receptor, primary biliary cholangitis, primary sclerosing cholangitis

## Abstract

**Purpose of review:**

This review analyses the main features of primary biliary cirrhosis (PBC) and primary sclerosing cholangitis (PSC) and provides an overview of the currently available (bile acid) bile acid related treatments.

**Recent findings:**

In PBC, biliary injury is the consequence of a dysregulated intrahepatic and systemic immune response. Given the close association between PSC and inflammatory bowel disease (IBD), the microbiota represents an important factor in the development of PSC. Bile acid based pharmacological treatments could represent promising therapeutic strategies in the management of cholangiopathies.

**Summary:**

Cholangiopathies include a spectrum of diseases resulting in cholestasis, an impairment of bile flow in the biliary tree, leading to biliary obstruction and damage as well as liver inflammation and fibrosis. PSC and PBC are highly heterogeneous cholangiopathies and progressive disorders with defined pathophysiological mechanisms. Curative treatments have not been established, and although their prevalence is low, they are a frequent indication for liver transplantation in the advanced stages of cholangiopathies. These diseases still present with unmet therapeutic strategies, also taking into account that on average 30–40% of patients undergoing liver transplantation will have recurrence of the original illness.

## INTRODUCTION

Cholangitis is a life-threatening condition defined by biliary obstruction and bacterial infiltration of the biliary tree [1]. First described in 1877 by Jean-Martin Charcot, the disease is characterized by fever, right upper quadrant pain and jaundice [2]. If not promptly treated, cholangitis can quickly progress into multiorgan dysfunction and death. Primary biliary cholangitis (PBC) and primary sclerosis cholangitis (PSC) represent the main immune-mediated chronic cholestatic liver diseases in adults that lead to liver cirrhosis or liver failure. Currently, there are not definitive curative treatments, and although their prevalence is low, they are a frequent indication for liver transplantation in the advanced stages of cholangiopathies. In the decade 1998--2008, 10% of all indications for liver transplantation in the USA were represented by PBC and PSC (https://optn.transplant.hrsa.gov/data/view-data-reports/national-data/). Novel therapeutic approaches encompass the use of transcriptional modifiers of bile formation. This review will analyse the main features of PBC and PSC and provide an overview of the currently available bile acid treatments. 

**Box 1:**
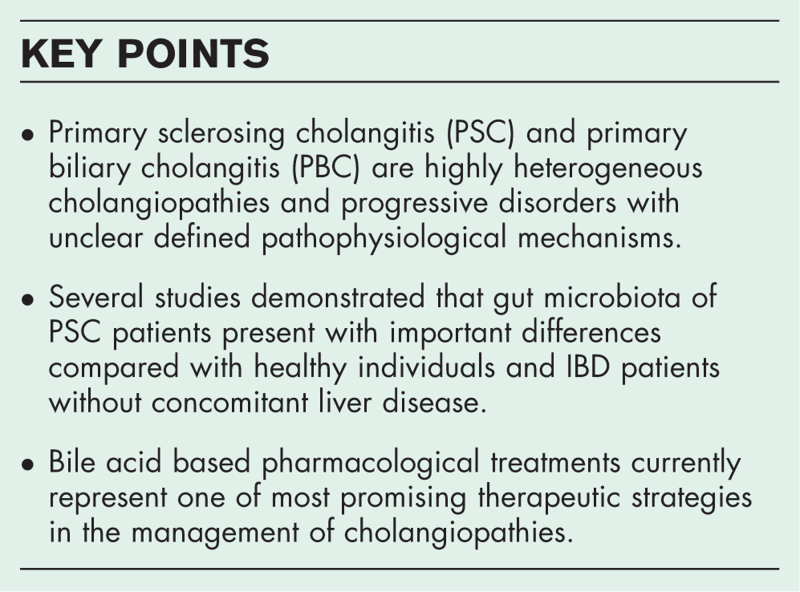
no caption available

### Clinical manifestation and molecular pathogenesis of the two main type of cholangitis: primary biliary cholangitis and primary sclerosing cholangitis

PBC and PSC are both progressive chronic cholestatic liver diseases. PBC is characterized by granulomatous destruction of small intrahepatic ducts [3], whereas PSC is defined by inflammation and fibrosis of the intrahepatic and extrahepatic bile ducts that promote bile duct stenoses [4,5]. Both PSC and PBC are characterized by an autoimmune trigger that leads to bile duct damage, cirrhosis and ultimately liver failure [6].

### Primary biliary cholangitis

PBC is caused by a combination of genetic predisposition – affecting T-cell regulation, extra-hepatic autoimmune diseases and PBC/positive antimitochondrial auto-antibody (AMA) [7–10] – and environmental factors, such as recurrent urinary tract infections, exposure to toxic chemicals and cigarette smoking [9]. PBC mainly affects middle-aged women and different patients present with different rates of advancement; however, it commonly progresses to terminal stages over 15–20 years. Serologic hallmarks of PBC include high alkaline phosphatase (ALP) and the presence of AMA [11]. The main symptoms of PBC encompass fatigue and pruritus especially at night inducing sleep disturbances and depression [12]. PBC patients can also be affected by skin lesions, lipid dysmetabolism, osteopenia/osteoporosis, hepatosplenomegaly, muscle wasting and oedema as a cirrhosis manifestation [13,14]. Cirrhosis can in turn, increase the risk of hepatocellular carcinoma development [15]. At biliary epithelial cell level, immune dysregulation is a typical feature of PBC due to the loss of tolerance to the E2 subunit of the mitochondrial pyruvate dehydrogenase complex (PDC-E2) [16]. Normal biliary epithelial cells are characterized by proper bicarbonate production contributing to the acidic environment at the surface of the biliary epithelium [17]. The anion exchanger 2 (AE2) is the principal bicarbonate exchanger regulating intracellular pH and biliary bicarbonate secretion leading to the peculiar bicarbonate-rich umbrella on the apical surface of cholangiocytes. The bicarbonate-rich umbrella is fundamental for biliary epithelial cells because it protects them from toxic hydrophobic bile acids. In fact, a dysfunctional AE2 leads to sensitization of biliary epithelial cells to apoptosis. Accumulation of senescent biliary epithelial cells present MHC class II molecules as well as several co-stimulatory inflammatory factors (TNF-α, IL-6, MCP-1, RANTES) collectively promoting an adaptive immune response [18]. Inflammatory cells enter into the epithelium leading to ductal luminal irregularities and epithelial interruption [19]. In the liver of PBC patients, natural killer T cells facilitate biliary epithelial cell damage, autoantigen release and activation of reactive T cells [20]. Plasma cells produce disease-specific AMAs that target immunodominant epitopes on PDC-E2 on the inner mitochondrial membrane ultimately contributing to cellular injury [21^▪^]. CD4^+^ T cells and CD8^+^ T cells are the main inflammatory cells within the portal tract and promote biliary damage [22]. Advanced fibrosis stages have been associated with an upregulation of pro-inflammatory Th17 cells that are necessary to support B-cell specific antibody production [23,24] and a downregulation of intrahepatic T reg cells and T follicular regulatory cells [25]. Furthermore, the biliary epithelium expresses toll like receptors (TLRs) that promote cellular injury via the secretion of pro-inflammatory molecules, such as IL-8 and CX3CL1, and recruitment of immune cells into the portal tract [26]. In this scenario, biliary injury is the consequence of a dysregulated intrahepatic and circulating immune response. Progressive bile duct deterioration results in impaired bile secretion and hepatic accumulation of bile acids.

### Primary sclerosing cholangitis

PSC is a progressive cholangiopathy that affects young men and is strongly linked to IBD [27]. The cause of PSC is still unclear, but it has been demonstrated an association with HLA-DRB1 and HLA-DQB1 haplotypes [28] and genes of the interleukin-2 pathway such as CD28 [29,30]. At the time of diagnosis, a high fraction of patients is asymptomatic [31]. Typical manifestations encompass fever and upper abdominal quadrant pain and can be accompanied by fatigue, pruritus and jaundice. These symptoms are due to inflammatory and cholestatic process promoting fibrosis and cirrhosis [32]. Furthermore, these patients present with hepatosplenomegaly, gallbladder disease, fat soluble vitamin malabsorption, metabolic bone disease and oesophageal varices, hematemesis and ascites as a consequence of portal hypertension [33]. IBD comorbidity varies and does not always associate with liver symptoms; however, patients presenting with both diseases have an increased risk of colorectal cancer onset compared with IBD patients without concomitant PSC and the general population [34]. Last but not least, PSC represents a risk factor for colangiocarcinoma [35]. The gold standard for the diagnosis of PSC is the cholangiogram because biochemical tests’ results may vary and do not correlate with disease progression [36]. The only treatment option for PSC patients is liver transplantation, but a high incidence of acute cellular rejection as well as PSC recurrence and IBD intensification have been shown [37].

Given the close association between PSC and IBD, the microbiota represents an important factor in its pathogenesis [38,39]. The liver and intestine are able to communicate with each other via the systemic circulation, portal vein and biliary tract. Intestinal inflammation or infections damage the intestinal epithelial barrier thereby allowing the translocation of microbes and pathogen-associated molecular patterns (PAMPs). Microbes and PAMPs, subsequently, reach the liver and activate hepatic immune cells (Kupffer and hepatic stellate cells) and, in turn, the production of pro-inflammatory cytokines, collectively leading to portal fibrosis and PSC [40,41]. Several studies demonstrated that the gut microbiota of PSC patients is different compared with healthy individuals and IBD patients without concomitant liver disease [42–44]. Quraishi *et al.*[45^▪^] demonstrated that microbial alterations and differentially expressed genes in PSC-IBD patients compared with IBD patients were due to a dysregulation of BAs metabolism in PSC-IBD patients. In PSC patients, dysbiosis translates into a reduction in bacterial diversity, an increased abundance of *Enterococcus, Fusobacterium, Lactobacillus* and *Veillonella* genera and a reduced abundance *Prevotella* and *Roseburia* species. The increased presence of *Enterococcus* is paralleled by increased ALP levels [46], mucosal inflammation and increased intestinal permeability and in bile. Abundance of *Enterococcus gallinarum* is associated with T helper 17 cells activation [47,48], while *Fusobacterium* correlates with intestinal inflammation severity [46]. Also, it has been demonstrated that *Veillonella* is associated with inflammatory and fibrotic conditions such as pulmonary fibrosis and PBC [49,50]. *Prevotella* and *Roseburia* species are butyrate producers and support the intestinal barrier function as well as the differentiation of regulatory T cells [51]; therefore, their decreased abundance in PSC patients is an additional burden. Furthermore, it has been observed that faecal microbiota of patients with PSC and concomitant IBD display an altered composition of fungal population characterized by an increased abundance of *Exophila* (a fungi genus involved in infections in immunodeficiency patients) and a decreased presence of *Saccaromyces cerevisiae*, which has been shown to have anti-inflammatory properties [52].

Interestingly, faecal microbiota transplantation (FMT) has been carried out in 10 patients with PSC and IBD in remission and it caused a strong reduction of ALP levels in 30% of patients, which was accompanied by increased bacterial diversity [53]. In 2019, two clinical trials have been interrupted because FMT caused bacteraemia with a drug-resistant *Escherichia coli* causing the death of one patient [54] and FMT has not been trialled anymore since then in PSC patients.

### Bile acids and the gut-liver axis

Despite the molecular difference of PBC and PSC, they both are mainly characterised by cholestasis, resulting from impaired bile formation or flow. Bile acids homeostasis is impaired in patients affected by cholangiopaties and given the prominent role of the intestine in this respect, understanding the gut-liver axis and bile acids physiology is crucial to recognize the concept behind the main bile acid based therapeutic strategies currently available for the clinical management of cholestatic liver diseases. Bile acids are detergent-like molecules synthesized in the liver and released after food ingestion into the small intestine wherein they aid the absorption of lipids and liposoluble nutrients. Hepatic de-novo bile acids synthesis is the result of cholesterol catabolism and involves the action of several enzymatic reactions, whose rate-limiting enzyme is the Cholesterol-7α-hydroxylase (Cyp7a1) transforming cholesterol intermediate metabolites into the two main primary bile acids: chenodeoxycholic (CDCA) and cholic acid [55]. In order to increase their water solubility and decrease their cytotoxicity, primary bile acids undergo a conjugation process with either glycine or taurine and are then secreted into bile together with cholesterol and phospholipids [56–58]. Conjugated bile acids are temporarily stored in the gallbladder and after food ingestion, the hormone cholecystokinin is then released from the proximal intestinal tract thereby stimulating the gallbladder to release bile into the duodenum, wherein the bile acids journey starts. Due to their detergent-like properties, bile acid facilitates the absorption of lipids and liposoluble vitamins along the small intestine. However, for the same reason, their levels must be kept in a tight range. In fact, abnormally elevated bile acids are highly cytotoxic, while their insufficient levels could cause a decrease in bile flow and consequent cholesterol supersaturation in bile that may contribute to the formation of cholesterol gallstone [59].

Bile acids synthesis is energetically costly; therefore, bile acids are efficiently re-circulated between the intestine and the liver in the so-called enterohepatic circulation. In fact, once they reach the terminal ileum 95% of bile acids are reabsorbed and travel via the portal vein back to the liver. Only 5% escape this recycling route, travel through the colon and are then excreted into faeces. The enterohepatic circulation and bile acid metabolism are modulated by nutrition and metabolic hormones. Metabolic nuclear receptors are crucial in this modulation, because they sense the available nutrients introduced with the diet or metabolites (including bile acids) produced according to nutritional inputs and prime the transcription of genes and hormones that orchestrate it. In particular, the Farnesoid X receptor (FXR) is a nuclear receptor and transcription factor highly expressed in the liver and intestine, activated by bile acids and is the master regulator of their homeostasis. FXR exploits its function with tissue-specific activities. In particular, at intestinal level, FXR promotes bile acids transport within the enterocytes, inducing IBABP [60–62], and their basolateral secretion into the portal circulation, inducing OSTα/β [63]. Apical enterocytes reabsorption via ASBT and sinusoidal hepatic re-uptake from the portal blood via NTCP present with species-specific differences and their precise regulation is still under debate, however in most instances FXR decreases ASBT expression [64]. Also, FXR induces the expression of the hepatic canalicular bile acid transport protein ABCB11 [57] and phospholipid floppase ABCB4 [65]. Moreover, FXR activation promotes the expression of BA-CoA-amino acid N-acetyltransferase (BAAT) and bile acid CoA synthase (BACS) [66], the two enzymes responsible of bile acids conjugation. As a result, cytotoxicity of detergent bile acid molecules in the biliary tract is prevented. Apart from regulating BAs transport, FXR regulates their synthesis and metabolism. In particular, at intestinal levels, FXR induces the expression of the fibroblast growth factor 15/19 (FGF15/19, 15 in mouse and 19 in humans), a peculiar family member of the FGF family, that acts as a metabolic hormone. Once produced, FGF15/19 is immediately secreted into the portal circulation and reaches the liver, where it binds to FGFR4-β Klotho (KLB) co-receptor heterodimer starting a phosphorylation cascade ultimately inhibiting Cyp7a1 expression, hence bile acids synthesis. Bile acids synthesis is also subjected to hepatic control, a mechanism that works in synergy with the intestinal one. Sophisticated experimental models have shown that the intestinal FXR and FGF19 is the predominant duo for the regulation of Cyp7a1 [67–69], while the hepatic FXR-SHP duo importantly controls KLB expression [70] with the aim of optimizing FGF19 action.

### Bile acid based pharmacological treatment

Cholangiopathies still present with unmet therapeutic strategies, also taking into account that on average 30–40% of patients undergoing liver transplantation will have recurrence of the original illness [71]. In addition, patients affected by cholangiopathies have an increased risk of developing cholangiocarcinoma [72^▪^].

### Primary biliary cholangitis

Until 2016, URSO has been the only approved drug for the clinical management of PBC. However, as for PSC, over 30% of patients do not achieve a sufficient hepatic biochemical response defined as a reduction in the surrogate biomarker ALP to less than 40% [73]. Evidence suggests that 13–15 mg/kg/day in divided doses is the most beneficial dose of UDCA in PBC patients. Lower or higher doses do not seem to display benefits when compared to the intermediate one [74], while much higher doses (up to 30 mg/kg/day) have shown to be harmful in patients with PSC [75]. In the 2016, FDA approved the use of obethicholic acid (OCA) -- a transcriptional modifier of bile formation and a strong activator of the bile acid sensor FXR -- as a second therapy for PBC patients in combination with URSO or in those who are unresponsive to UDCA [76,77]. Currently, a phase IV study on OCA is ongoing and evaluating clinical outcomes and hard primary end points including hepatic decompensation, transplant and death in PBC patients (the COBALT study, clinicaltrials.gov NCT02308111). As seen in clinical studies testing FXR agonists in PSC, the most common adverse event in PBC is dose-dependent mild to moderate pruritus [76,77]. In addition to OCA, the effects of two other FXR agonists: tropifexor and EDP-305 are being trialed. A multipart, double blind clinical study to assess safety, tolerability and efficacy of tropifexor has recently been completed (clinicaltrial.gov NCT02516605) and results are awaited. After recently published promising results about the efficacy of EDP-305 in a mouse model of preestablished biliary fibrosis and steatohepatitis [78^▪▪^], a clinical trial to assess safety, tolerability, pharmacokinetics and efficacy of EDP-305 has been completed and results are anticipated.

### Primary sclerosing cholangitis

To date, no therapeutic strategy has been proven to be successful in arresting PSC progression and current options focus on the management of symptoms, such as pruritus and fatigue and comorbidities, such as ulcerative colitis usually with a pancolitis phenotype, autoimmune diseases, metabolic bone diseases and bacterial cholangitis. Clinical endoscopic management of biliary strictures [79] and the use of antibiotics have been key strategies for handling the majority of complications. In particular, given its immunomodulatory action and ability to increase the production of antimicrobial peptides, oral vancomycin has been used, especially to manage IBD-associated PSC [80]. In a recently completed small clinical trial, oral vancomycin has been shown to improve liver biochemistry, it was well tolerated and no patient displayed treatment adverse event [81]. However, despite showing promising results, formal recommendations are not available yet [82] and further clinical trials are ongoing (clinicaltrials.gov NCT03710122). One of the most extensively studied therapeutic agents for PSC is Ursodeoxycholic acid (UDCA), a secondary bile acid with cytoprotective activities given his high hydrophilicity. UDCA has anticholestatic effects, stimulates BAs and organic anion secretion and the secretion of biliary bicarbonate at hepatocytes and cholangiocytes level leading to the stabilization of the biliary umbrella [83–85]. URSO is also believed to have a beneficial influence on the immune system with its anti-inflammatory properties and reduces the severity of cell injury [86]. Different clinical trials have been performed, and it has been shown that UDCA may slow down PSC progression at some level; however, a real clinical effectiveness has yet to be demonstrated [84,85]. It has been shown that at a moderate dose (13–15 mg/kg/day) in some PSC patients reduces liver enzymes (e.g. ALP) compared with placebo; however, no significant difference in clinical endpoints has been achieved. Currently, there are no consensus guidelines [e.g. the American Society of Hepatology Clinical Practice Guidelines (2010), the American College of Gastroenterology Guidelines (2015) and the European Hepatology Society Clinical Practice Guidelines (2009)] regarding the use of low to moderate doses of UDCA in PSC [87]. After a promising phase II study result [88], a phase 3 study is ongoing (clinicaltrials.gov NCT03872921) testing the use of 24-norursodeoxycholic acid (norUDCA). NorUDCA is a side chain-shortened C23 homologue of UDCA and a synthetic bile acid, producing a bile acid dependent bicarbonate-rich choleresis. Also, other studies are ongoing testing the efficacy of UDCA in combination with all-trans retinoic acid [89].

Novel therapeutic approaches encompass the use of OCA. Recently, a randomized, placebo-controlled, phase II clinical study of OCA in PSC was completed. This study has shown that treatment with 5–10 mg of OCA reduced serum ALP in patients with PSC during an initial 24-weeks treatment period. Mild to moderate dose-related pruritus was the most common adverse event. The result was sustained during the following 2-year, long-term extension of the study [90^▪▪^]. Another FXR agonist, Cilofexor (aka GS-9674) has also been tested in a phase II double-blind, placebo-controlled study for 12 weeks. Cilofexor was well tolerated and led to a dose-dependent significant improvement in liver biochemistries and markers of cholestasis in patients with PSC, with pruritus as the most common adverse event [91]. Intriguingly, given the role of the FXR-FGF19 duo in controlling bile acids synthesis and homeostasis, also an analogue of FGF19 has been recently tested in clinical trials for PSC. Aldafermin (aka NGM282) is an engineered version of FGF19 that display its bile acids homeostasis regulatory properties but lack its potential pro-tumorigenic activity [92]. Aldafermin has been tested in clinical trials to treat PSC patients. A phase 2 study has shown that after 12 weeks of treatment there were no significant changes in serum ALP levels from baseline between the Aldafermin and placebo groups (primary endpoint), despite significant reduction in markers of cholestasis and fibrosis in the treatment group [93].

## CONCLUSION

Cholangiopathies continue to have high mortality and pose significant challenges for their clinical management. Apart from the ones described in this review, there are also other therapies currently being developed or trialled. One of them is the clinical use of fibrates, targeting peroxisome proliferator-activated receptors (PPARs). PPARs are three nuclear receptors (α, β and δ) also partially involved in the regulation of BAs homeostasis. Fenofibrate and bezafibrate are among some of the most promising emerging therapies for PBC [94–97]. Moreover, there are studies on the use of immunomodulators with and without concomitant use of UDCA (e.g. budesonide [98–101], methotrexate [102–105], mycophenolate [106–108], mAb or modulator of T-cell activity or recruitment [109,110], agents targeting oxidative stress and inflammation [111]). However, studies are still small and clinical trial design and execution are held back by several factors, including the heterogeneity of clinical presentation and progression, the rarity of these diseases and the difficult decision about clinically relevant endpoints. Although a number of molecular mechanisms and genetic abnormalities typically involved in the pathogenesis of cholangiopathies are being unravelled, significant points are still obscure and environmental contributors are largely unknown. This clearly influences the generation of knowledge translation into clinical therapies and transversal applicability of the current results to patients is difficult. UDCA remains the cornerstone therapy of cholangiopathies, despite other abnormalities characterize the pathogenesis and progression of these disease (e.g. immune problems and dysbiosis).

## Acknowledgements

*None.*

### Financial support and sponsorship

*This work was supported by AIRC IG 2019 23239 to A.M.*

### Conflicts of interest

*There are no conflicts of interest.*

## REFERENCES AND RECOMMENDED READING

Papers of particular interest, published within the annual period of review, have been highlighted as:

▪ of special interest
▪▪ of outstanding interest
